# Oxidatively Modified LDL Suppresses Lymphangiogenesis via CD36 Signaling

**DOI:** 10.3390/antiox10020331

**Published:** 2021-02-23

**Authors:** Bhupesh Singla, Hui-Ping Lin, WonMo Ahn, Joseph White, Gábor Csányi

**Affiliations:** 1 Vascular Biology Center, Medical College of Georgia, Augusta University, Augusta, GA 30912, USA; hlin@augusta.edu (H.-P.L.); wahn@augusta.edu (W.A.); 2 Department of Pathology, Medical College of Georgia, Augusta University, Augusta, GA 30912, USA; jwhite3@augusta.edu; 3 Department of Pharmacology and Toxicology, Medical College of Georgia, Augusta University, Augusta, GA 30912, USA

**Keywords:** oxidized LDL, native LDL, lymphangiogenesis, atherosclerosis, CD36

## Abstract

Arterial accumulation of plasma-derived LDL and its subsequent oxidation contributes to atherosclerosis. Lymphatic vessel (LV)-mediated removal of arterial cholesterol has been shown to reduce atherosclerotic lesion formation. However, the precise mechanisms that regulate LV density and function in atherosclerotic vessels remain to be identified. The aim of this study was to investigate the role of native LDL (nLDL) and oxidized LDL (oxLDL) in modulating lymphangiogenesis and underlying molecular mechanisms. Western blotting and immunostaining experiments demonstrated increased oxLDL expression in human atherosclerotic arteries. Furthermore, elevated oxLDL levels were detected in the adventitial layer, where LV are primarily present. Treatment of human lymphatic endothelial cells (LEC) with oxLDL inhibited in vitro tube formation, while nLDL stimulated it. Similar results were observed with Matrigel plug assay in vivo. CD36 deletion in mice and its siRNA-mediated knockdown in LEC prevented oxLDL-induced inhibition of lymphangiogenesis. In addition, oxLDL via CD36 receptor suppressed cell cycle, downregulated AKT and eNOS expression, and increased levels of p27 in LEC. Collectively, these results indicate that oxLDL inhibits lymphangiogenesis via CD36-mediated regulation of AKT/eNOS pathway and cell cycle. These findings suggest that therapeutic blockade of LEC CD36 may promote arterial lymphangiogenesis, leading to increased cholesterol removal from the arterial wall and reduced atherosclerosis.

## 1. Introduction

Atherosclerotic vascular disease is the underlying cause of stable and unstable angina, myocardial infarction, stroke, and peripheral artery disease [1]. Atherosclerosis is a chronic inflammatory disease characterized by the influx, oxidation, and accumulation of apoB-containing lipoproteins in large and medium-sized arteries. Recent studies have demonstrated the importance of arterial lymphatic vessels (LV) in the removal of cholesterol via HDL-mediated reverse cholesterol transport, egress of inflammatory cells from the arterial wall and regulation of arterial inflammation [2,3]. Furthermore, genetic disruption of lymphatic drainage using soluble vascular endothelial growth factor receptor 3 (sVGFR3) overexpression, which results in LV regression, and surgical removal of lesion-draining lymph nodes promote atherosclerosis development in hypercholesterolemic mice [2,4,5]. Consistent with this information, a recent study showed that disruption of aortic lymph flow by LV ligation stimulates atherosclerotic lesion formation [3]. The same study reported that regression of atherosclerosis induced by cholesterol lowering therapy in ApoE^−/−^ mice requires efficient lymphatic drainage. Taken together, these results suggest that a functional network of LV in the arterial wall and periadventitial layer is required to inhibit the development of atherosclerosis and promote plaque regression. Despite this information, the precise endogenous factors and mechanisms that regulate LV formation and function relevant to atherosclerosis development are largely unknown.

In the early phase of atherosclerosis, circulating low-density lipoprotein (LDL) is transported across the endothelial layer of the arterial wall and becomes oxidized due to the pro-inflammatory and oxidative microenvironment in the subendothelial space. The oxidized LDL (oxLDL) plays a pathogenic role in all stages of atherosclerosis development, including initiation, progression, and destabilization of atherosclerotic lesion [6,7,8]. Availability of oxLDL antibodies developed by immunizing animals with malondialdehyde-modified LDL, 4-hydroxynonenal-LDL, and Cu^2+^-oxidized LDL has allowed determination of oxLDL levels in the plasma and arterial tissue from cardiovascular disease patients [9,10]. Elevated plasma oxLDL levels were detected in patients with atherosclerotic artery disease [11,12]. Furthermore, clinical and experimental studies showed evidence suggesting the presence of oxidatively modified LDL in atherosclerotic arteries [11,13,14]. In addition, increased circulating LDL levels in ApoE−/− mice has been shown to regress LV density [15,16]. Nevertheless, the ability of oxLDL to regulate lymphangiogenesis has not been previously reported.

Multiple reports suggest that oxLDL contributes to endothelial cell activation and dysfunction, macrophage foam cell formation, vascular smooth muscle cell migration, proliferation and differentiation to macrophage-like cells, as well as platelet adhesion and aggregation [8,17,18,19]. Interestingly, in vitro studies demonstrated biphasic effects of oxLDL treatment on angiogenesis. At low concentrations, oxLDL stimulates angiogenesis, however, at pathophysiologically relevant concentrations found in the plasma of cardiovascular patients, it suppresses angiogenesis [9,20,21,22]. Oxidized LDL can be internalized by various scavenger receptors (SR), including CD36, SR-A1, SR-B1, and lectin-like oxLDL receptor 1 (Lox1), and has reduced affinity for LDL receptor [23]. Lymphatic endothelial cells (LEC) mainly utilize fatty acids via mitochondrial fatty acid oxidation for their energy needs and have higher expression of fatty acid transport proteins, such as CD36 [24]. Though, angiogenesis and lymphangiogenesis share similar regulatory mechanisms, the effects of oxLDL on lymphangiogenesis, involvement of specific oxLDL receptor(s), and underlying molecular mechanisms have not been previously investigated.

In the present study, we sought to examine the ability of unmodified native and oxidized LDL in the regulation of lymphangiogenesis using various in vitro cellular, molecular, histological, and pharmacological approaches and in vivo genetic models. First, we detected elevated oxLDL levels in the adventitial layer and intraplaque regions of human atherosclerotic arteries. We observed increased lymphangiogenesis by nLDL-treated human LEC, while oxLDL treatment inhibited lymphangiogenesis both in vitro and in vivo. Mechanistically, we found that oxLDL induces cell cycle arrest in LEC and inhibits lymphangiogenesis via CD36. Downstream regulators of oxLDL-induced inhibition of lymphangiogenesis include decreased AKT and eNOS protein expression and increased cyclin dependent kinase inhibitor p27 levels. Taken together, these findings demonstrate for the first time the anti-lymphangiogenic effects of oxLDL and identify the possible downstream mechanisms involved. These results suggest that therapeutic blockade of LEC CD36 may promote lymphangiogenesis in the arterial wall, leading to increased removal of arterial cholesterol and delayed development of atherosclerosis.

## 2. Materials and Methods

### 2.1. Reagents and Antibodies

Human nLDL and oxLDL were purchased from Kalen Biomedical, LLC (Germantown, MD, USA). BLT1 was procured from Sigma-Aldrich (St. Louis, MO, USA). Growth factor-reduced (GFR) Matrigel was obtained from Corning (Bedford, MA, USA). FxCycle™ PI/RNase Staining Solution, DAF-FM diacetate, Hoechst 33342, H2DCFDA, and DAPI were purchased from Life Technologies Corporation (Eugene, OR, USA). Antibody against human oxLDL was procured from Biorbyt LLC (St. Louis, MO, USA). Phospho-eNOS (Ser-1177), phospho-AKT (Ser-473), phospho-ERK1/2 (Thr-202/Tyr-204), total AKT, total ERK1/2, p27, CD36, and β-tubulin antibodies were obtained from Cell Signaling Technology (Danvers, MA, USA). Total eNOS, Ki67, p53, CDK1/2, and GAPDH antibodies were procured from Santa Cruz Biotechnology (Dallas, TX, USA). LYVE-1 antibody was purchased from Abcam (Cambridge, MA, USA). Protease and phosphatase inhibitor cocktail tablets were bought from Roche Diagnostics GmbH (Mannheim, Germany).

### 2.2. Human Atherosclerotic Tissue

Human atherosclerotic and non-atherosclerotic aortic and coronary artery tissue were obtained from three female (69–101 years old) and two male (57 and 88 years old) cadaveric donors. All females had atherosclerotic coronary artery disease and were Caucasians. Out of these females, one female had a history of systemic hypertension and the other female had aortic stenosis. Tissues were also collected from a Caucasian male with coronary artery disease and a non-atherosclerotic African-American male. The presence or absence of atherosclerotic lesions was confirmed by Joseph White, Director of Autopsy Services at Augusta University and Oil Red O staining. Additional information about the tissue donors, including cause of death, comorbidities, and race is available in our previous publication [25]. The study was approved by the Biological Safety Office, Augusta University (BSP# 1458) and conducted in accordance with the guidelines in the Declaration of Helsinki. The written informed consent was obtained on behalf of each case from next of kin, for use of postmortem tissue for research purposes.

### 2.3. Animals

Eight- to ten-week-old male C57BL/6 (wild type, JAX, stock # 000664) and CD36-deficient (CD36^−/−^, JAX, stock # 019006) mice were used in the present study. All mice were maintained on 12-h dark/12-h light cycles in air-conditioned rooms with access to food and drinking water ad libitum. All animal experimental procedures were approved by the Institutional Animal Care and Use Committee of Augusta University and conducted following the National Institutes of Health Guide for the Care and Use of Laboratory Animals.

### 2.4. Cell Culture

Primary human dermal lymphatic endothelial cells (LEC) were purchased from PromoCell GmbH (Heidelberg, Germany) and cultured in endothelial cell growth medium MV 2 (PromoCell) containing 5% heat-inactivated fetal bovine serum (FBS), growth factors bullet kit [human epidermal growth factor (5 ng/mL), human basic fibroblast growth factor (10 ng/mL), insulin-like growth factor (20 ng/mL), human vascular endothelial growth factor 165 (0.5 ng/mL), ascorbic acid (1 μg/mL), and hydrocortisone (0.2 μg/mL)], 100 IU/mL of penicillin, and 100 μg/mL streptomycin. Cells were maintained in a humidified incubator with 5% CO_2_ at 37 °C and used till passage 7.

Human umbilical vein endothelial cells (HUVEC) were obtained from ATCC (Manassas, VA, USA) and cultured in endothelial cell growth medium MV 2 (PromoCell) as described above.

### 2.5. Western Blot

Western blot experiments were performed using tissue/cell lysates as described previously using the Odyssey CLx Infrared Imaging System (Li-Cor Biosciences, Lincoln, NE, USA) [26]. Briefly, protein concentrations in lysate preparations were quantified employing the Pierce BCA Protein Assay Kit (ThermoFisher Scientific). Equal amounts of proteins (15–20 μg) were separated using 12% SDS-PAGE gels, transferred onto nitrocellulose membranes (Li-Cor Biosciences), and membranes were blocked with the Intercept Blocking Buffer (Li-Cor Biosciences). Blocked membranes were probed with the following primary antibodies: oxLDL, phospho-eNOS (Ser-1177), total eNOS, phospho-AKT (Ser-473), total AKT, phospho-ERK1/2 (Thr-202/Tyr-204), total ERK1/2, CD36, p53, p27, CDK1/2, GAPDH, and β-tubulin. The IRDye-conjugated secondary antibodies (Li-Cor Biosciences) were utilized to detect the primary antibodies bound to membranes. The densitometry analysis was performed using the NIH ImageJ software.

### 2.6. Immunohistochemistry

Lipid deposition in human aortic sections was determined using Oil Red O staining as previously described [25]. Briefly, harvested tissue was fixed in 4% PFA for 48 h, embedded in OCT compound and 8 µm sections prepared. Sections were washed with PBS to remove OCT and then incubated with 60% isopropanol, stained with Oil Red O solution (Sigma-Aldrich, 2.0% *w*/*v*), and counterstained with hematoxylin (Sigma-Aldrich). Images were taken using an Olympus BX41 phase contrast microscope.

Human arterial paraffin sections were deparaffinized, blocked, and incubated with primary antibodies against oxLDL overnight at 4 °C in a humidified chamber. Then, sections were washed with PBST 2 times, incubated with Alexa Fluor 488-conjugated secondary antibodies (Life Technologies Corporation) for 1 h at room temperature, washed, counterstained with DAPI, and mounted with Fluoromount-G (ThermoFisher Scientific, Rockford, IL, USA). Images were captured using an inverted Zeiss LSM 780 confocal microscope.

### 2.7. LEC Tube Formation Assay

In vitro lymphangiogenesis was determined using a Matrigel tube formation assay as previously described [25]. Briefly, LEC (20,000 cells/well) in basal medium MV 2 (PromoCell GmbH, 0.5% FBS) containing vehicle (PBS), nLDL (50 or 100 µg/mL) or oxLDL (50 or 100 µg/mL) were seeded onto solidified Matrigels in wells of a 96-well plate and incubated for 6 h at 37 °C in a humidified incubator. Matrigels were fixed, permeabilized, stained with Alexa Fluor 488-phalloidin, and images were recorded using an inverted Zeiss LSM 780 confocal microscope. The tube length and number of branching points were measured using the NIH ImageJ software.

### 2.8. LEC Migration Assay

LEC migration was investigated using Culture-Insert 2 Well 24 (ibidi USA, Inc., Fitchburg, WI, USA). Briefly, 70 μL of the cell suspension (3 × 10^5^ cells/mL) was applied into each well of the Culture-Inserts 2 Well. The plate was incubated for 24 h at 37 °C in 5% CO_2_. Culture-Inserts 2 Well were removed, and cell layers washed with PBS 2 times and treated with vehicle, nLDL (100 µg/mL) or oxLDL (100 µg/mL) in basal medium MV 2 (0.5% FBS) for 24 h at 37 °C. Images of wounds were captured using an Olympus BX41 phase contrast microscope at 0 h. After 24 h of incubation, cell layers were washed, fixed, permeabilized, and stained with Alexa Fluor 488-phalloidin. Images of wounds were taken using an inverted Zeiss LSM 780 confocal microscope. Area of wounds at 0 h and 24 h were determined using the Image-Pro Plus software (Media Cybemetics, Bethesda, MD, USA). The percentage of wound closure was calculated.

### 2.9. LEC Proliferation Assays

For determination of Ki67 positive cells, LEC were plated on coverslips inserted in a 24-well plate. LEC were treated with vehicle, nLDL (100 µg/mL) or oxLDL (100 µg/mL) for 24 h. Cells were fixed, permeabilized, blocked, and incubated with Ki67 primary antibodies overnight at 4 °C. Cells were then incubated with Alexa Fluor 594-labeled secondary antibodies (Life Technologies Corporation) and Hoechst 33342. Images were captured using an inverted Zeiss LSM 780 confocal microscope. Ki67 positive nuclei were quantified using the NIH ImageJ software.

LEC proliferation was measured using the Cell Proliferation Reagent WST-1 (Roche Diagnostics GmbH). Briefly, cells were seeded in a 96-well plate (10,000 cells/well). Next day, cells were incubated with vehicle, nLDL (100 µg/mL) or oxLDL (100 µg/mL) in basal medium MV 2 containing 0.5% FBS for 48 h. Following 48 h treatment, 10 μL WST-1 reagent was added to each well, and cells were incubated at 37 °C for 4 h. The absorbance was measured at 450 nm using the Clariostar Monochromator Microplate Reader (BMG Labtech, Cary, NC, USA). Absorbance at 690 nm was taken as reference.

### 2.10. Cell Cycle Analysis

Cells after 24 h treatment with vehicle, nLDL or oxLDL (100 µg/mL) were washed with PBS and fixed with ice-cold 70% ethanol for 2 h at 4 °C. After washing with cold PBS, cells were incubated in FxCycle™ PI/RNase Staining Solution for 30 min in dark at room temperature. The samples were immediately analyzed by flow cytometry using a BD Accuri C6 flow cytometer.

### 2.11. In Vivo Lymphangiogenesis Assay

In vivo lymphangiogenesis was determined using the Matrigel plug assay [23]. Eight- to ten-week-old male wild type or CD36^−/−^ mice on the C57BL/6 background were injected subcutaneously (s.c.) with 400 µL GFR Matrigel premixed with vehicle (PBS), nLDL (100 µg/mL) or oxLDL (100 µg/mL). Matrigel plugs were harvested after 2 weeks of implantation, fixed in 4% paraformaldehyde (PFA), embedded in paraffin blocks, and sectioned. Paraffin sections were deparaffinized, blocked, and processed for LYVE-1 immunostaining. Fluorescent images were captured using a Zeiss 780 inverted confocal microscope. Image fluorescence analysis was performed with Image-Pro Plus software.

### 2.12. Quantitative Real-Time PCR

Total cellular RNA was extracted utilizing an RNA purification kit (IBI Scientific, Peosta, IA, USA). Five hundred nanogram RNA was reverse transcribed with TaqMan^®^ Reverse Transcriptase kit (Applied Biosystems, Carlsbad, CA, USA) as per the manufacturer’s instructions. SYBR Green Supermix (Applied Biosystems) was used to perform quantitative real-time PCR in a StepOne Plus system (Applied Biosystems). Relative gene expression was determined with the ΔΔ*C*_t_ method using GAPDH (human/mouse) as an internal control. A standard real-time PCR protocol (one cycle of 95  °C for 10  min and 40 cycles of 95  °C for 15  s and 60  °C for 1 min) was used for all reactions using different primers. The primer sequences used for real-time PCR are shown in Supplementary Table S1.

### 2.13. Gene Silencing

Human LEC were transfected with CD36 siRNA (smart pool of siRNA) or control siRNA (Horizon Discovery, St. Louis, MO, USA) using the TransIT-TKO transfection reagent (Mirus Bio LLC, Madison, WI, USA) according to the manufacturer’s instructions. Gene silencing using siRNA was confirmed by Western blot. Cells were used for further experiments 48 h post-transfection.

### 2.14. Lipid Internalization Assay

Control and CD36-siRNA treated cells were plated in a 24-well plate. The next day, cells were incubated with or without ox-LDL (100 μg/mL) for 24 h. After 24 h, cells were washed with ice-cold PBS, fixed in 2% PFA and stained with Nile Red (50 ng/mL) for 7 min. Fluorescence intensity was measured using the FL3 channel for Nile Red (Ex: 488 nm, Em: 670 nm; BD Accuri C6).

### 2.15. Reactive Oxygen Species Generation

Intracellular reactive oxygen species (ROS) production was measured using 2′,7′-dichlorodihydrofluorescein diacetate (H2DCFDA) [27]. Briefly, LEC seeded in a 12-well plate were treated in the presence and absence of oxLDL for various time points. After the incubation time, cells were washed with serum free media twice and incubated in media containing with H2DCFDA (5 μM, 30 min) at 37 °C. The flow cytometry analysis (Ex: 492 nm, Em: 525 nm) was performed using a BD Accuri C6 flow cytometer. Mean fluorescence intensity is shown.

### 2.16. Statistical Analysis

The statistical analyses were performed using the GraphPad Prism (La Jolla, CA, USA). The data are expressed as mean ± SEM. Shapiro–Wilk normality test was used to investigate the normality of data. Student’s *t*-test and 1 or 2-way ANOVA were used, followed by Tukey post hoc analysis for multiple comparisons where appropriate. A *p*-value less than 0.05 was considered statistically significant.

## 3. Results

### 3.1. Oxidized LDL Levels Are Elevated in Human Atherosclerotic Arteries

Subendothelial accumulation of LDL and its subsequent oxidative modification play a critical role in the pathomechanisms of atherosclerosis [28]. Oxidized LDL promotes endothelial cell activation and dysfunction [8], macrophage lipid uptake and foam cell formation [19], and vascular smooth muscle cell migration [18], proliferation and transdifferentiation to macrophage-like cells [29]. The role of oxLDL in the adventitial layer of arterial tissue is less characterized. To investigate the expression of oxLDL in the intimal, medial and adventitial layers of atherosclerotic arteries, we collected human atherosclerotic aortic and coronary artery tissue and non-atherosclerotic control segments from cadaveric donors with and without a history of cardiovascular disease.

**Figure 1:**
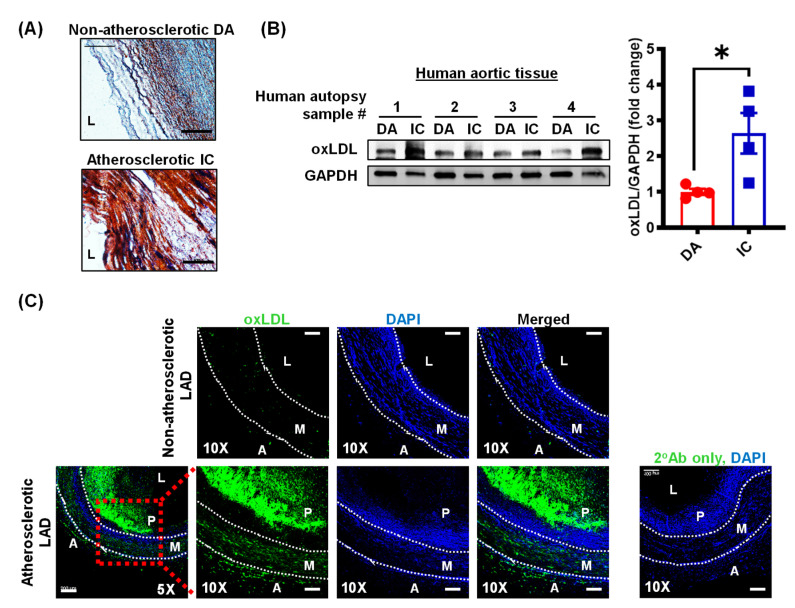
Oxidized LDL levels are increased in human atherosclerotic arteries. (**A**) Human aortic tissues (IC and DA) isolated from cadaveric donors were stained with Oil Red O to identify atherosclerotic lesions. Representatives of *n* = 4 experiments shown. Scale bar: 100 µm. (**B**) Human aortic tissue lysates were subjected to Western blot analysis for oxLDL and GADPH expression. Representative Western blot images are shown. Bar graph represents mean protein levels along with individual data points calculated using densitometric analysis and expressed as a ratio of oxLDL to GAPDH (*n* = 4). (**C**) Atherosclerotic and non-atherosclerotic human LAD coronary arteries were immunostained for oxLDL (green) and nuclei were counterstained with DAPI (blue). Representative images are shown (*n* = 3 for atherosclerotic LAD and *n* = 1 for non-atherosclerotic control LAD). Scale bar: 200 µm (leftmost panel). Magnified images of red inset are also shown, scale bar: 100 µm. Data represent the mean ± SEM. * *p* < 0.05. DA: descending aorta; IC: inner curvature; LAD: left anterior descending; L: lumen; A: adventitia; M: media; and P: plaque.

Atherosclerosis is a focal disease that occurs in regions of low shear stress and disturbed flow, including bifurcations and curvatures. Oil Red O staining confirmed lipid accumulation in the inner curvature (IC) of the aortic arch compared to the atheroresistant descending thoracic aorta (DA) (Figure 1A). Western blot experiments demonstrated increased oxLDL levels in human atherosclerotic IC tissue compared to non-atherosclerotic regions of the aorta (Figure 1B).

Next, immunostaining experiments were performed to investigate levels of oxLDL in the lesion area, medial layer and adventitia of atherosclerotic left anterior descending (LAD) coronary arteries. Consistent with the immunoblotting data, we observed increased oxLDL expression in the plaque (P) area and medial layer (M) of coronary arteries compared with non-atherosclerotic coronary artery tissue (Figure 1C). Similarly, oxLDL staining was increased in the adventitial layer (A) of human atherosclerotic LAD (Figure 1C). Taken together, these data demonstrate that oxLDL levels are increased in all layers of human atherosclerotic arteries.

### 3.2. Oxidized LDL Suppresses Lymphangiogenesis In Vitro and In Vivo

**Figure 2:**
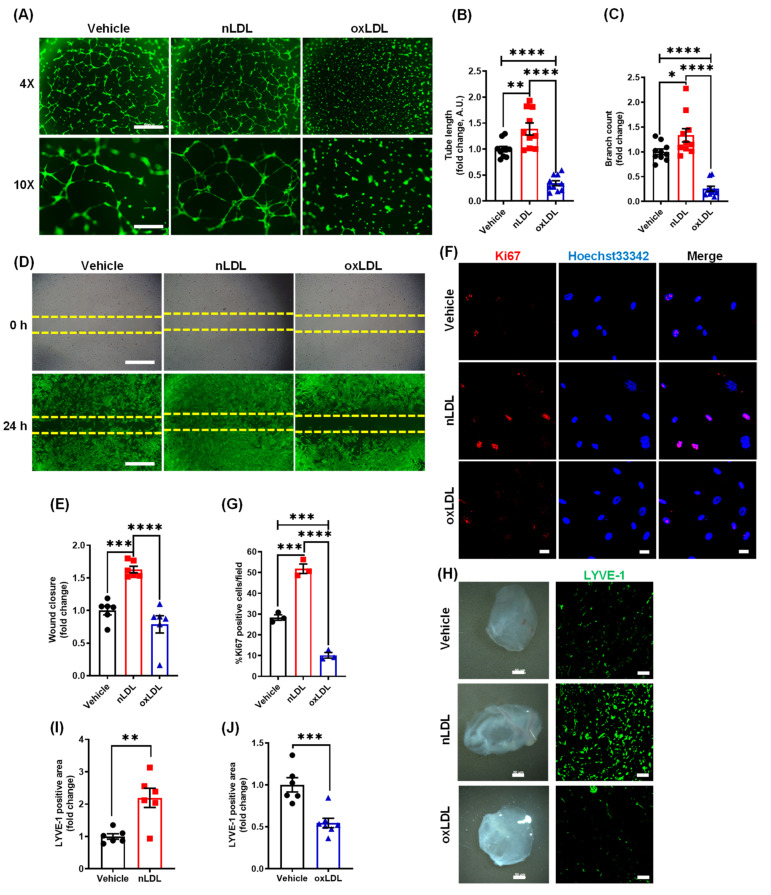
Oxidized LDL suppresses lymphangiogenesis in vitro and in vivo. (**A**–**C**) Human LEC in basal media MV2 (0.5% FBS) containing vehicle (PBS), nLDL (100 µg/mL) or oxLDL (100 µg/mL) were seeded in wells of a Matrigel-coated plate and tube formation determined after 6 h. (**A**) Representative images for LEC tube formation assay are shown. Scale bars: 500 μm for 4× magnification and 200 μm for 10× magnification. Images of random fields were taken, and tube length (**B**) and the number of branching points (**C**) quantified (*n* = 10). All experiments were performed in duplicate. (**D**,**E**) LEC migration in response to vehicle, nLDL (100 µg/mL) or oxLDL (100 µg/mL) was investigated after 24 h using Culture-Insert 2 Well 24 (ibidi USA). (**D**) Representative images of wounds at 0 h and 24 h are shown. Scale bar: 500 μm. **(E)** Quantification of wound closure (*n* = 6). (**F**,**G**) LEC grown on coverslips were treated with vehicle, nLDL, or oxLDL for 24 h. Cells were fixed and immunostained for Ki67 (red). Nuclei were counterstained with Hoechst 33342 (blue). Images were captured from more than five random fields. (**F**) Representative images of Ki67 immunostaining. Scale bar: 20 μm. (**G**) Bar graph represents the mean number of Ki67 positive cells/field (*n* = 3). (**H**–**J**) Wild-type mice were injected s.c. with Matrigel solutions premixed with vehicle, nLDL, or oxLDL. Matrigel plugs were harvested after 2 weeks of implantation. (**H**) Representative images of harvested Matrigels and LYVE-1 staining of the cross-sections of the Matrigel plugs are shown. Scale bar: 50 μm. (**I**,**J**) Quantification of LYVE-1 positive area for **H** (*n* = 6). Data represent the mean ± SEM. * *p* < 0.05; ** *p* < 0.01; *** *p* < 0.005; and **** *p* < 0.001.

Previous studies demonstrated that lymphatic vessels are primarily expressed in the adventitial layer of large and medium-sized arteries and disruption of lymphatic drainage promotes atherosclerotic lesion formation [4,25,30]. Therefore, we next examined whether oxLDL treatment regulates lymphangiogenesis in vitro and in vivo using a Matrigel-based tube formation assay and Matrigel plug assay, respectively. Human LEC were incubated with vehicle, control lipoprotein nLDL (100 µg/mL) and oxLDL (100 µg/mL) for 6 h. As shown in Figure 2A–C, nLDL stimulated while oxLDL treatment inhibited tube length and number of branching points compared with vehicle-treated controls. Similar findings were observed when LEC were treated with 50 µg/mL concentration of nLDL or oxLDL (Supplementary Figure S1). Wound healing assay data indicated that nLDL stimulated LEC migration (24 h), however oxLDL challenge did not significantly affect wound closure compared with vehicle (Figure 2D,E). Next, we performed immunofluorescence experiments to quantify the number of Ki67 positive nuclei following vehicle-, nLDL-, or oxLDL-treatment to assess LEC proliferation (24 h). Immunofluorescence analysis showed a significantly increased number of Ki67 positive nuclei in nLDL-treated LEC compared with vehicle-treated cells (Figure 2F,G). On the contrary, oxLDL treatment reduced the number of Ki67 positive nuclei compared with both vehicle and nLDL treatment (Figure 2F,G). Taken together, these findings suggest that unmodified nLDL stimulates, while oxidatively modified LDL inhibits lymphangiogenesis in vitro.

Next, the Matrigel plug lymphangiogenesis assay was used to quantify newly formed LV in implanted gel plugs in vivo. Vehicle, nLDL, or oxLDL-containing Matrigel solutions were injected (s.c.) into wild type (C57BL/6J) mice. Two weeks after implantation, Matrigel plugs were isolated and processed for LYVE-1 immunostaining to determine LV density. Immunostaining data revealed a remarkably increased LYVE-1 positive area in nLDL-containing plugs compared with vehicle controls (Figure 2H,I).

On the other hand, oxLDL-containing plugs showed decreased LYVE-1 positive staining indicating inhibition of in vivo lymphangiogenesis by oxLDL treatment (Figure 2H,J). Consistent with our in vitro observations, these data indicate the anti-lymphangiogenic and pro-lymphangiogenic potential of oxLDL and nLDL in vivo, respectively.

### 3.3. Pharmacological Blockade of SR-B1 Does Not Rescue OxLDL-Induced Inhibition of Lymphatic Endothelial Cell Proliferation and Tube Formation

**Figure 3:**
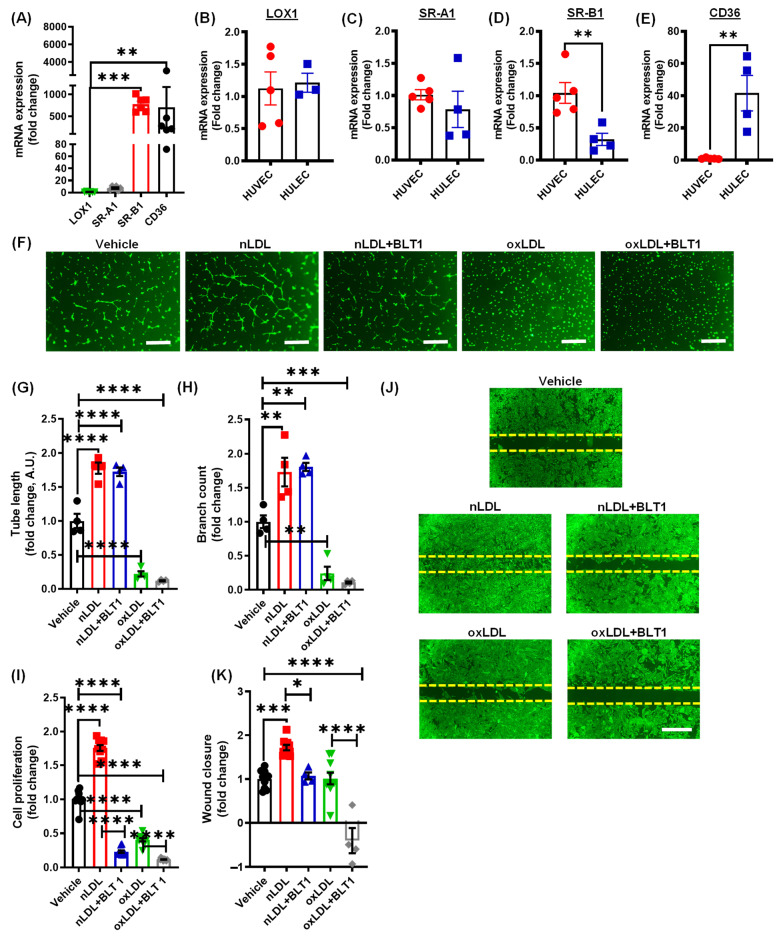
Pharmacological blockade of SR-B1 does not rescue oxLDL-induced inhibition of lymphatic endothelial cell proliferation and tube formation. (**A**) RNA isolated from human dermal LEC was used to determine the relative mRNA expression of LOX-1, SRA1, SRB1 and CD36. GAPDH was used as internal control. Bar graph represents mRNA levels in comparison to LOX1 (gene with the lowest expression) (*n* = 6). (**B**–**E**) comparison of mRNA expression of LOX-1 (**B**), SRA1 (**C**), SRB1 (**D**), and CD36 (**E**) in human LEC (HULEC) and human venous endothelial cells (HUVEC) (*n* = 4, 5). (**F**–**K**) LECs were pretreated with vehicle or BLT-1 (10 µM, 1 h), then incubated with vehicle (PBS), nLDL, or oxLDL and tube formation (6 h), cell proliferation (48 h), and cell migration (24 h) investigated as described in Figure 2. (**F**) Representative images for LEC tube formation assay are shown. Scale bar: 250 μm. Tube length (**G**) and branching points (**H**) are quantified (*n* = 4). (**I**) Quantification of LEC proliferation using the WST-1 assay (*n* = 10). (**J**,**K**) Representative images of wounds after 24 h and quantification (*n* = 4–10). Scale bar: 500 μm. Data represent the mean ± SEM. * *p* < 0.05; ** *p* < 0.01; *** *p* < 0.005; and **** *p* < 0.001.

LOX1, SR-A1, SR-B1, and CD36 have been described as the primary scavenger receptors for oxLDL in various cell types, including macrophages, smooth muscle cells and endothelial cells [23]. To identify the receptor(s) involved in the oxLDL-induced inhibition of lymphangiogenesis, we first carried out qRT-PCR experiments to determine the relative mRNA expression of LOX1, SR-A1, SR-B1, and CD36 in human LEC. The qRT-PCR data identified SR-B1 and CD36 as the major oxLDL receptors in LEC (Figure 3A). Quantitative RT-PCR analysis was also performed to compare the expression of these oxLDL receptors between human LEC and human vascular endothelial cells HUVEC (Figure 3B–E). Interestingly, we observed higher levels of CD36 mRNA and lower levels of SR-B1 mRNA in LEC compared to HUVEC (Figure 3D,E).

To examine the role of SR-B1 in oxLDL-induced inhibition of lymphangiogenesis, LEC were treated with the SR-B1 inhibitor BLT1 prior to incubation with oxLDL [31]. As shown in Figure 3F–H, BLT1 treatment did not attenuate the inhibitory effect of oxLDL on LEC tube length and number of branching points. Similarly, oxLDL-induced inhibition of cell proliferation was not prevented by SR-B1 blockade (Figure 3I). A previous study demonstrated the role of the LEC SR-B1 receptor in the removal of cholesterol from peripheral tissue [32]. Consistent with these results, we found that nLDL-induced stimulation of LEC proliferation and wound closure was inhibited by BLT1 (Figure 3I–K). Interestingly, BLT1 did not inhibit nLDL-induced stimulation of LEC tube formation (Figure 3F–H). Finally, exposure to BLT1 alone inhibited LEC proliferation in the absence of exogenous LDL (Supplementary Figure S2), indicating the importance of SR-B1 in LDL-independent LEC proliferation. These data demonstrate that blockade of SR-B1-mediated signaling does not prevent oxLDL-mediated inhibition of lymphangiogenesis, but it may play a role in nLDL-stimulated LEC proliferation and migration.

### 3.4. Oxidized LDL Mediates Its Anti-Lymphangiogenic Effect via CD36

**Figure 4:**
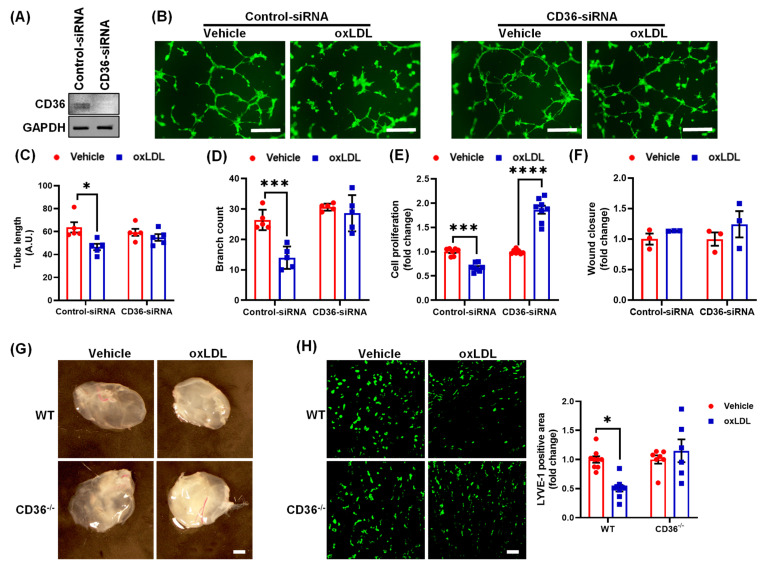
Oxidized LDL inhibits lymphangiogenesis via CD36. (**A**–**F**) Human LEC were transfected with control-siRNA or CD36-siRNA. After 48 h, cells were treated with vehicle or oxLDL. (**A**) Western blot analysis of CD36 protein expression in control and CD36 siRNA-treated LEC. (**B**) Representative images of LEC tube formation. Scale bar: 200 μm. (**C**) Quantification of tube length (*n* = 5). (**D**) Quantification of branch count (*n* = 5). (**E**) Cell proliferation analysis in control and CD36-silenced LEC using the WST-1 assay (*n* = 7–8). (**F**) Quantification of cell migration following vehicle and oxLDL treatment (*n* = 3). (**G**–**H**) Wild type (WT) and CD36^−/−^ mice were injected subcutaneously (s.c.) with Matrigel solutions premixed with vehicle or oxLDL. (**G**) Representative images of harvested Matrigel plugs. Scale bar: 50 μm. (**H**) LYVE-1 immunostaining. Scale bar: 50 μm. Bar graph shows the quantification of LYVE-1 positive area (*n* = 7–9). Data represent the mean ± SEM. * *p* < 0.05; *** *p* < 0.005; and **** *p* < 0.001.

To determine the role of CD36 in oxLDL-mediated inhibition of lymphangiogenesis, siRNA-mediated knockdown of CD36 was performed in LEC. Representative western blot experiments demonstrate a successful knockdown of CD36 in LEC (Figure 4A). As shown in Figure 4B–D, oxLDL inhibited both tube length and branch point formation in control siRNA-treated cells but not in CD36-silenced cells. Furthermore, treatment with oxLDL significantly suppressed the proliferation of control LEC, which was rescued by CD36 silencing (Figure 4E). The wound healing experiments indicated no statistical differences in migration between control and CD36-silenced LEC treated with oxLDL (Figure 4F). Next, WT and CD36^−/−^ mice implanted with Matrigel plugs were used to investigate the mechanisms by which oxLDL inhibits lymphangiogenesis in vivo. Matrigel plugs were harvested 2 weeks after implantation (Figure 4G) and LYVE-1 staining was performed on cross-sections of isolated Matrigels. Immunofluorescence data showed that oxLDL significantly inhibited LYVE-1 positive area in WT mice, but this inhibitory effect of oxLDL was attenuated in CD36^−/−^ mice (Figure 4H). A control experiment was performed to investigate whether oxLDL is internalized by LEC via binding to cell surface CD36 receptor. Nile Red flow cytometry data showed decreased lipid accumulation in oxLDL-treated CD36-silenced cells compared with control cells, confirming the role of CD36 in oxLDL internalization by LEC (Supplementary Figure S3) [33]. Collectively, these findings suggest that CD36 mediates oxLDL-induced inhibition of lymphangiogenesis.

### 3.5. Oxidized LDL Induces Cell Cycle Arrest and Inhibits AKT and eNOS Expression in LEC

**Figure 5:**
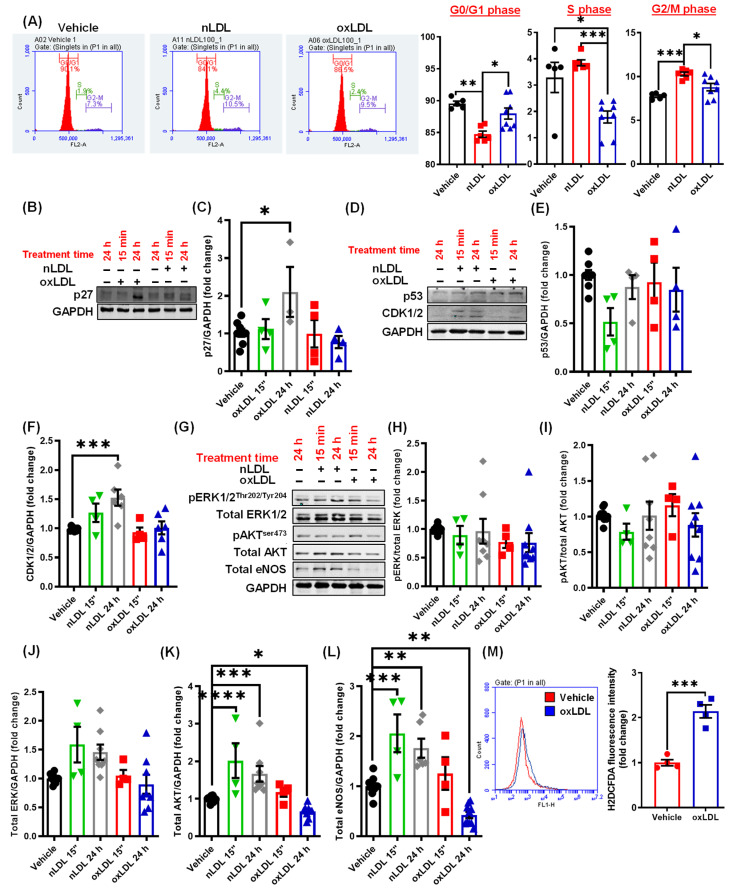
Oxidized LDL induces cell cycle arrest and inhibits AKT and eNOS expression in LEC. (**A**) Human LEC were treated with vehicle, nLDL, or oxLDL for 24 h, and cell cycle analysis was performed using flow cytometry. Representative flow cytometry histograms for each treatment are shown. Bar graphs show quantification of cell cycle (cell percentage, *n* = 5–8). (**B**–**L**) LEC were treated with vehicle, nLDL, or oxLDL for 15 min or 24 h and cell lysates were subjected to Western blot analysis of various proteins, including p27 (**B**,**C**), p53 (**D**,**E**) and CDK1/2 (**D**,**F**), pERK (**G**,**H**), pAkt (**G**,**I**), total ERK (**G**,**J**), total AKT (**G**,**K**) and total eNOS (**G**,**L**). (*n* = 4–7). (**M**) LEC were treated with vehicle or oxLDL for 15 min and H2DCFDA fluorescence determined using flow cytometry. Representative histograms indicating H2DCFDA fluorescence are shown. The *x*-axis is logarithmic. Bar diagram indicates mean fluorescence intensities (*n* = 4). Data represent the mean ± SEM. * *p* < 0.05; ** *p* < 0.01; *** *p* < 0.005; and **** *p* < 0.001.

Next, we investigated the mechanisms by which oxLDL downstream of CD36 inhibits lymphangiogenesis. Consistent with the ability of oxLDL to inhibit cell proliferation, flow cytometry analysis demonstrated a decreased number of LEC in the S-phase of cell cycle following oxLDL treatment (Figure 5A), suggesting cell cycle arrest. In contrast, nLDL-treated cells showed an increased percentage of cells in the G2/M phase compared with vehicle (Figure 5A). Next, we investigated the molecular basis underlying this alteration in the cell cycle following oxLDL and nLDL treatment [34]. Cyclin dependent kinase (CDK) 1/2, p27 and p53 play important roles in the regulation of cell cycle [35,36]. Increased CDK1/2 induces cell cycle progression, while elevated expression of p27, a CDK inhibitor, leads to cell cycle arrest [36]. Activation or upregulated expression of p53 is crucial for initiating senescence in response to DNA damage [37]. Therefore, we next determined the levels of these proteins in LEC following nLDL and oxLDL treatments. Treatment with oxLDL stimulated expression of CDK inhibitor p27 (Figure 5B,C), while nLDL treatment upregulated CDK1/2 expression (Figure 5D,F) in LEC after 24 h. No differences in p53 levels were detected after oxLDL or nLDL treatment compared with vehicle (Figure 5D,E).

The PI3K-AKT-eNOS signaling pathway is an important and well-known positive regulator of lymphangiogenesis [38]. Furthermore, AKT has been shown to promote cell cycle progression by inducing the expression of various cyclins and inhibiting the expression of multiple negative regulators of cell cycle, including p27, p21, and p15 [36,39]. As we found increased p27 expression in oxLDL-treated cells (Figure 5B), we determined whether AKT/eNOS pathway is also suppressed by oxLDL treatment. Immunoblotting data demonstrated that nLDL stimulates both AKT and eNOS protein expression in LEC (Figure 5G,K,L). On the other hand, oxLDL reduced AKT and eNOS expression after 24 h of treatment (Figure 5G,K,L). No significant differences in total ERK1/2 protein levels were found between control and LDL-treated cells (Figure 5G,J). Moreover, the phosphorylation status of ERK1/2 and AKT were not different between treatment groups (Figure 5G–I).

Previous studies have demonstrated the role of oxidative stress in the suppression of lymphangiogenesis [25,27]. Therefore, we examined whether oxLDL treatment induces intracellular reactive oxygen species (ROS) production in LEC using the H2DCFDA fluorescence method. As shown in Figure 5M, oxLDL-treated cells generated significantly higher amounts of ROS compared with vehicle-treated controls. Altogether, these results suggest that oxLDL promotes p27 (an inhibitor of cell cycle) expression possibly via downregulating AKT-mediated signaling and stimulates ROS production in LEC.

### 3.6. CD36 Knockdown Attenuates the Inhibitory Effects of oxLDL on Cell Cycle

**Figure 6:**
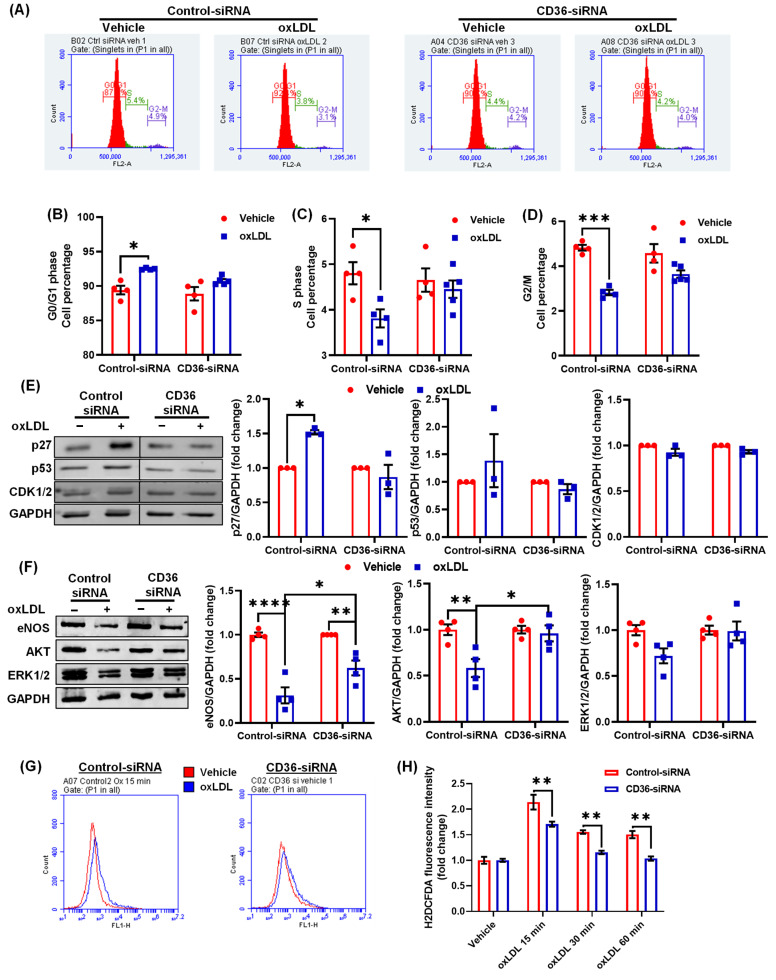
CD36 silencing rescues inhibitory effects of oxLDL on cell cycle**.** Human LEC were transfected with control or CD36 siRNA. After 48 h, cells were treated with vehicle or oxLDL for 24 h. (**A**–**D**) Cell cycle analysis was performed using flow cytometry. (**A**) Representative flow cytometry histograms. Bar graphs show quantified number of cells in G0/G1 (**B**), S (**C**), and G2/M (**D**) phases (*n* = 4–5). (**E**) Cell lysates were used to determine p27, p53, and CDK1/2 protein expression (*n* = 3–4). **(F)** ERK, AKT and eNOS protein expression (*n* = 3, 4). (**G**,**H**) LEC were treated with vehicle or oxLDL for the indicated time points and H2DCFDA fluorescence analysed using flow cytometry. (**G**) Representative histograms indicating H2DCFDA fluorescence (15 min) are shown. The *x*-axis is logarithmic. (**H**) Bar diagram indicates mean fluorescence intensity (MFI) in vehicle- and oxLDL-treated cells (*n* = 4). Data represent the mean ± SEM. * *p* < 0.05; ** *p* < 0.01; *** *p* < 0.005; and **** *p* < 0.001.

Our cell cycle studies indicated a reduced number of cells in the S-phase of cell cycle following oxLDL treatment (Figure 5A). We next investigated the effects of CD36 silencing on cell cycle after oxLDL treatment. Interestingly, silencing of CD36 prevented oxLDL-induced cell cycle arrest as demonstrated by the similar percentage of CD36-siRNA treated cells in the G0/G1, S and G2/M phases with or without oxLDL treatment (Figure 6A–D). In addition, CD36 knockdown abrogated oxLDL-induced p27 expression in LEC, which was stimulated in control siRNA-treated cells (Figure 6E). Moreover, there were no differences in the levels of p53 and CDK1/2 proteins between control and CD36-silenced cells following oxLDL treatment (Figure 6E). Further, immunoblotting experiments demonstrated increased expression of eNOS and AKT proteins in CD36-silenced LEC compared with control-siRNA treated cells after oxLDL treatment (Figure 6F).

As treatment with oxLDL induced intracellular ROS generation in LEC (Figure 5M), we further evaluated ROS production in control and CD36-silenced cells by measuring H2DCFDA fluorescence at various time points after oxLDL stimulation. As shown in Figure 6G,H, CD36-silenced cells generated significantly less ROS after 15, 30 and 60 min of oxLDL stimulation compared with control cells. Taken together, these data suggest that deletion of CD36 in LEC prevents inhibition of cell cycle progression, increases expression of lymphangiogenic signaling proteins and reduces ROS production after oxLDL treatment.

## 4. Discussion

The lymphatic vasculature plays a critical role in immune cell trafficking and clearance of macromolecules, pathogens, cytokines and lipids from inflamed tissue. An increasing number of studies have demonstrated that low LV density and impaired lymphatic clearance contribute to pathological conditions, including atherosclerosis, hypertension, diabetes, obesity, chronic lung disease, renal fibrosis and edema development [25,40,41,42,43]. However, the molecular factors and underlying mechanisms regulating LV density and function in peripheral tissue, such as the atherosclerotic artery, are poorly understood. Atherosclerosis is associated with an accumulation of unmodified nLDL and its oxidative derivative, oxLDL, in arterial tissue. In this study, we investigated the ability of nLDL and oxLDL to regulate lymphangiogenesis in vitro and in vivo. Our data demonstrate that unmodified nLDL and oxLDL differently regulate lymphangiogenesis; nLDL stimulates, while oxLDL inhibits lymphangiogenesis. Furthermore, oxLDL inhibits cell cycle, increases levels of cell cycle inhibitor p27, and suppresses AKT and eNOS protein expression in LEC, leading to impaired lymphangiogenesis. CD36 gene silencing attenuates oxLDL-induced inhibition of lymphangiogenesis in vitro, consistent with increased LV density in oxLDL containing Matrigel plugs implanted into CD36^−/−^ mice in vivo. Taken together, these findings suggest that therapeutic blockade of LEC CD36 signaling may promote lymphangiogenesis, leading to increased clearance of inflammatory cells, removal of interstitial cytokines, and HDL-mediated arterial cholesterol efflux. This, in turn, may support the resolution of arterial inflammation and restoration of tissue homeostasis in atherosclerotic vessels.

Increased plasma LDL concentrations are associated with a higher rate of cardiovascular events [44]. Native LDL, which accounts for the majority of plasma LDL, is a heterogeneous class of lipoproteins consisting of a hydrophobic core containing triglycerides, cholesteryl esters, and free cholesterol, a hydrophilic shell of phospholipids and transmembrane apolipoproteins [45]. Native LDL binds to and is mainly internalized by the LDL receptor. Additionally, increased oxLDL levels have been found in the plasma of coronary artery disease patients and atherosclerotic lesion oxLDL levels correlate with plaque destabilization and rupture [9,11,12]. Systemic inflammation, hypertension, diabetes, and low shear stress play an important role in the development of endothelial dysfunction, which is a critical early event in the initiation of atherosclerosis. Cholesterol functions as a precursor molecule in the synthesis of steroid hormones and is an important lipid constituent of plasma membrane that regulates membrane fluidity and receptor function [46,47]. Importantly, changes in circulating LDL levels do not always correspond to pathological changes in the arterial wall [48]. A study by Kromhout et al. reported significantly different 25-year cardiovascular mortality despite similar levels (200 mg/dL) of serum cholesterol, suggesting a complex association between arterial lipid accumulation, diet, modifiable and non-modifiable cardiovascular risk factors, genetic susceptibility and mortality [49]. In addition, Libby et al. suggested that inflammation provides a mechanistic link among the traditional risk factors for atherosclerosis and pathophysiological alterations occurring in the vessel wall [50]. Therefore, a combination of anti-inflammatory therapy and lipid-lowering agents may be used in cardiovascular disease patients as supported by the results obtained from the CANTOS trial [51].

The lymphatic vasculature is regarded as the primary route of cholesterol removal from atherosclerotic arteries via reverse cholesterol transport and inhibition of lymphatic drainage exacerbates atherosclerosis [2,4]. However, the physiological factors and precise molecular mechanisms that regulate LV density and function in the arterial wall remain to be identified. Our immunoblotting and immunostaining experiments demonstrated increased expression of oxLDL in human atherosclerotic arteries compared to non-atherosclerotic control tissue. Further, oxLDL positive areas were mainly detected in the macrophage-rich intraplaque region and adventitial layer of human atherosclerotic coronary arteries. These results are consistent with those of Uchida et al. who showed the presence of oxLDL in the intima, adventitia and periadventitial adipose tissue of human atherosclerotic coronary arteries [10]. We would like to add that application-specific validation of antibody specificity is required to confirm our results and previous reports showing increased oxLDL levels in atherosclerotic vessels. As oxLDL levels seem elevated in the adventitial layer of atherosclerotic vessels, where LVs are mainly found [2], we next determined the effects of oxLDL treatment on lymphangiogenesis using in vitro LEC proliferation, migration, and tube formation assays and an in vivo Matrigel plug method. Our results indicated that oxLDL (100 µg/mL) inhibits lymphangiogenesis both in vitro and in vivo. Interestingly, we found that unmodified nLDL (100 µg/mL) stimulates lymphangiogenesis. Similar findings were observed when LEC were treated with a lower concentration of nLDL and oxLDL in vitro (50 µg/mL). Relevant to this information, Nishi et al. reported that plaque oxLDL levels are approximately 70 times higher than plasma oxLDL in cardiovascular disease patients [9]. However, the precise concentrations of oxLDL, extent of LDL oxidation, and specific lipid and apoprotein oxidation of lipoproteins in different anatomical regions and at different stages of plaque development are not known. Lymphatic endothelial cells mainly utilize fatty acids via mitochondrial fatty acid oxidation for their energy needs. Fatty acid oxidation has been shown to promote LEC proliferation as it provides acetyl-CoA, which helps to sustain the Krebs cycle and dNTP synthesis for cell division [24]. These data support our findings of increased LEC proliferation after nLDL treatment in vitro and stimulated LV density in Matrigel plugs transplanted into WT mice in vivo. In addition, a recent study utilizing mass spectrometry demonstrated a notable decrease in the metabolites of mitochondrial Krebs cycle in LEC following oxLDL treatment [52]. These results are consistent with the reduced proliferation of oxLDL-treated LEC observed in the present study. Interestingly, oxidized LDL treatment did not inhibit LEC migration in vitro. It is, however, possible that oxLDL-treated LEC may have shown reduced migration under conditions that stimulate lymphangiogenesis (e.g., VEGF-C or fibroblast growth factor 2 treatment [53]). It would be also interesting to investigate migration toward a gradient of soluble chemoattractants in control- and oxLDL-challenged LEC using the μ-slide chamber assay. It is also possible that oxLDL may have an effect on migration distance without influencing the direction of cell movement.

Several scavenger receptors for oxLDL have been reported in various cell types, including macrophages, smooth muscle cells and endothelial cells. These include LOX1, SR-A1, SR-B1, and CD36 [23]. We next quantified the levels of various oxLDL receptors in human LEC. Quantitative PCR data identified SR-B1 and CD36 as major oxLDL receptors in LEC. The higher levels of SR-B1 in LEC are consistent with the role of initial LV in the removal of cholesterol from peripheral tissue [32]. Indeed, free cholesterol loaded-HDL particles bind to LEC SR-B1 receptors and undergo transcytosis across the LEC layer. A recent study by Huang et al. demonstrated that SR-B1 promotes endothelial cell transcytosis of plasma nLDL and contributes to atherosclerosis development [31]. Furthermore, endothelial cell-specific SR-B1 deletion attenuates atherosclerosis. To investigate the role of SR-B1 in the anti-lymphangiogenic effect of oxLDL, we used BLT1 a small molecule inhibitor of SR-B1 [31]. Our data demonstrated that BLT1 does not prevent oxLDL-induced inhibition of lymphangiogenesis, but it suppresses nLDL-stimulated LEC proliferation and migration. It is important to add that LDLR mRNA expression is to the levels of SR-B1 and CD36 in LEC (Supplementary Figure S4). Future studies using LDLR knockout LEC are required to investigate the role of LDLR in nLDL-induced lymphangiogenesis.

As SR-B1 inhibition did not rescue oxLDL-induced suppression of lymphangiogenesis, engagement of CD36 in this process was evaluated. CD36 is also called fatty acid translocase. It binds to many ligands including thrombospondin-1, long chain fatty acids, collagen type I, nLDL and oxLDL [54]. CD36 levels were also detected higher in LEC, which support the importance of FAO in LEC as described above [24]. Global deletion of CD36 in murine models has been shown to reduce atherogenesis [55]. The protective effect of CD36 deletion, however, has been attributed to inhibition of macrophage oxLDL uptake and reduced foam cell formation. CD36 silencing in LEC demonstrated that oxLDL attenuates lymphangiogenesis via CD36 signaling. In addition, in vivo Matrigel plug assay performed using CD36^−/−^ mice confirmed these in vitro findings. These data are consistent with inhibition of oxLDL uptake by CD36-silenced LEC. Importantly, a previous study identified an association between CD36 gene single nucleotide polymorphisms (SNPs) and decreased atherosclerosis [56], however, no data are available regarding the effects of these SNPs on lymphangiogenesis or lymphatic function. Moreover, adventitial LV density in hypercholesterolemic CD36^−/−^ mice has not been investigated.

Mechanistically, we found that oxLDL treatment induced cell cycle arrest, increased p27 protein levels, and reduced expression of pro-lymphangiogenic mediators, including AKT and eNOS via CD36-dependent pathways. AKT has been shown to promote cell cycle progression by inducing the expression of various cyclins and inhibiting the expression of cell cycle inhibitors such as p27, p21, and p15 [36,39]. The mechanism by which oxLDL reduces AKT expression in LEC is unclear. Previous studies have shown increased autophagy in vascular smooth muscle cells and vascular endothelial cells following oxLDL treatment [57,58,59]. Furthermore, activation of autophagy is known to reduce the levels of AKT protein in cancer cells [60]. It is possible that oxLDL stimulates autophagy in LEC, which leads to decreased AKT expression. In addition, Zamora et al. suggested a negative association between lymphangiogenesis and autophagy [61]. Future studies are required to investigate the effects of oxLDL treatment on autophagy in LEC. Increased intracellular ROS production was found in oxLDL-treated LEC compared to control cells. These data are in line with a previous report indicating the inhibitory effects of oxidative stress on lymphangiogenesis via VEGFR3 degradation [27]. Furthermore, ROS have been shown to induce cell cycle arrest in endothelial cells [62]. A recent study indicated that increased proliferation of LEC with PI3K mutation is associated with AKT hyperactivation [63]. Nonetheless, whether AKT overexpression or hyperactivation in LEC and scavenging of ROS can prevent oxLDL-induced suppression of lymphangiogenesis remain to be investigated. In addition, future studies using LEC-specific CD36 knockout mice are required to determine the role of LEC CD36 in the regulation of arterial LV density, reverse cholesterol transport and development of atherosclerosis. In this study, human dermal LEC were utilized to investigate the effects of oxLDL/nLDL on lymphangiogenesis. Although these cells are widely used to investigate LEC intracellular signaling and lymphangiogenesis [38,64], it may be possible that LEC forming arterial LVs respond differently to oxLDL/nLDL treatments.

Statins are widely used for treatment of hypercholesterolemia. Statin treatment inhibits vascular endothelial cell proliferation and sprouting [65,66]. Schulz et al. investigated the effects of four different statins including mevastatin, lovastatin, simvastatin, and atorvastatin on lymphangiogenesis, and found that all these statins inhibit growth factors- stimulated lymphangiogenesis in a concentration-dependent manner [67]. Moreover, atorvastatin has also been shown to suppress lymphangiogenesis [68]. However, the role of statins in regulating the effects of nLDL/oxLDL treatment on lymphangiogenesis has never been explored. Future studies are needed to investigate arterial lymphangiogenesis in cardiovascular disease patients with or without statin therapy.

## 5. Conclusions

In summary, the present study demonstrates that nLDL stimulates and oxLDL inhibits lymphangiogenesis. The oxidative modification of LDL is responsible for the inhibition of lymphangiogenesis via CD36-dependent mechanisms. Treatment with oxLDL induces cell cycle arrest and reduces expression of AKT and eNOS in LEC. These findings may lead to future studies evaluating the therapeutic effect of LEC CD36 inhibition to promote arterial lymphangiogenesis, stimulate the removal of arterial cholesterol and attenuate atherosclerosis development.

## Data Availability

The data related to this article is contained within the article or Supplementary Material.

## Supplementary Materials

The following are available online at https://www.mdpi.com/2076-3921/10/2/331/s1, Figure S1: Oxidized LDL suppresses lymphangiogenesis in vitro. Figure S2: BLT1 treatment inhibits LEC proliferation. Figure S3: CD36 mediates oxLDL uptake by LEC. Figure S4: LDLR receptor expression in LEC. Table S1: List of primers used for mRNA quantitation using quantitative real-time PCR.
