# Supplementary material for: Oxidatively Modified LDL Suppresses Lymphangiogenesis via CD36 Signaling
Source: Antioxidants (Basel). 2021 Feb 23;10(2):331. doi: 10.3390/antiox10020331 (PMC7926875; doi:10.3390/antiox10020331)
Supplement: Supplementary file 1 [file antioxidants-10-00331-s001.pdf]

Supplementary Table 1: List of primers used for mRNA quantitation using quantitative real-time PCR.

Gene name	Primer sequences
**Human Lox1**	F 5'- CTC CTT TGA TGC CCC ACT TA -3'<br>R 5'- TTT CCG CAT AAA CAG CTC CT -3'
**Human SR-A1**	F 5'- GCA GTT CTC ATC CCT CTC AT -3'<br>R 5'- TCT TCG TTT CCC ACT TCA GG -3'
**Human SR-B1**	F 5'- TTT GAA GGC ATC CCC ACC TA -3'<br>R 5'- TGA ATT CCA GAC TCC AGG CAC -3'
**Human CD36**	F 5'- CTT TGG CTT AAT GAG ACT GGG AC-3'<br>R 5'- GCA ACA AAC ATC ACC ACA CCA-3'

Supplementary Figure S1

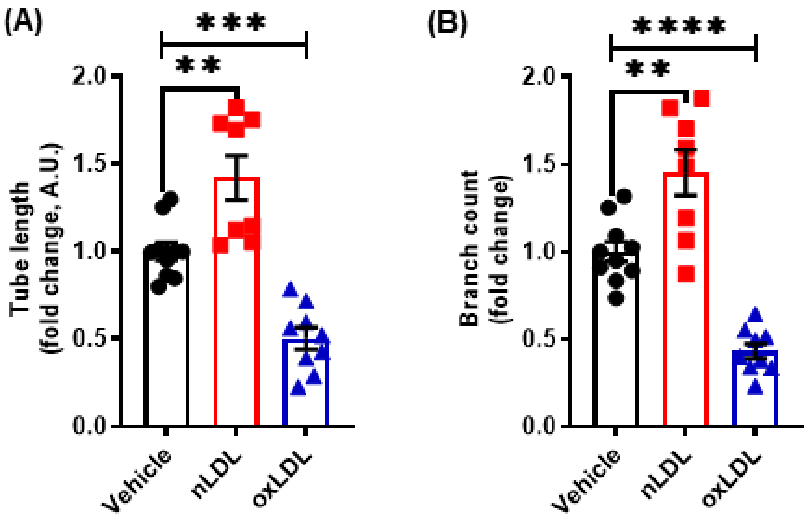

Supplementary Figure S1. Oxidized LDL suppresses lymphangiogenesis *in vitro*. (A-B) Human LEC in basal media MV2 (0.5% FBS) containing vehicle, nLDL (50 µg/mL) or oxLDL (50 µg/mL) were seeded in wells of a Matrigel-coated plate and tube formation determined after 6 h. Qunatification of tube length ***(A)*** and the number of branching points ***(B)*** are shown (n = 8-10). Data represented the mean ± SEM. **$p < 0.01$; *** $p < 0.005$.

Supplementary Figure S2

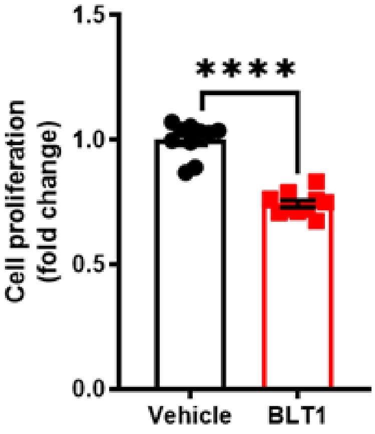

Supplementary Figure S2. BLT1 treatment inhibits LEC proliferation. Human LEC were treated with vehicle or BLT1 (10 µM) for 48 h and cell proliferation determined using WST-1 assay (*n* = 10). Data represented the mean ± SEM. **** $p < 0.001$.

Supplementary Figure S3

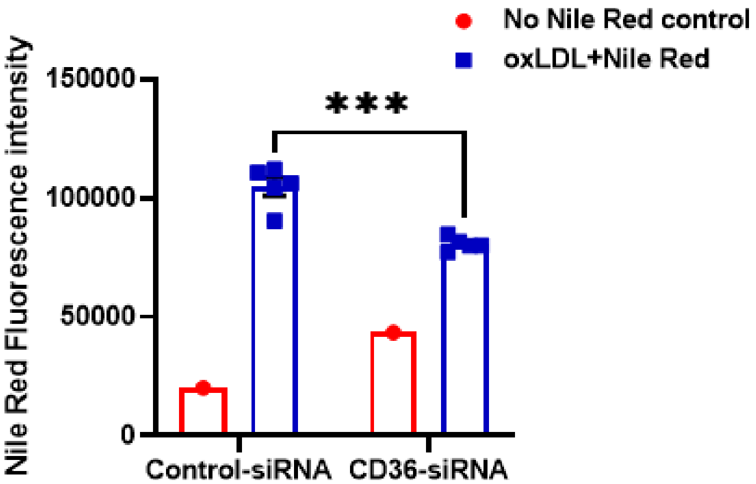

Supplementary Figure 3. CD36 mediates oxLDL uptake by LEC. Human LEC were transfected with control- or CD36-siRNA. After 48 h, cells were treated with oxLDL for 24 h. Cells were stained with Nile Red (50 ng/mL, 7 min) and FACS analysis performed to determine lipid uptake.

Supplementary Figure S4

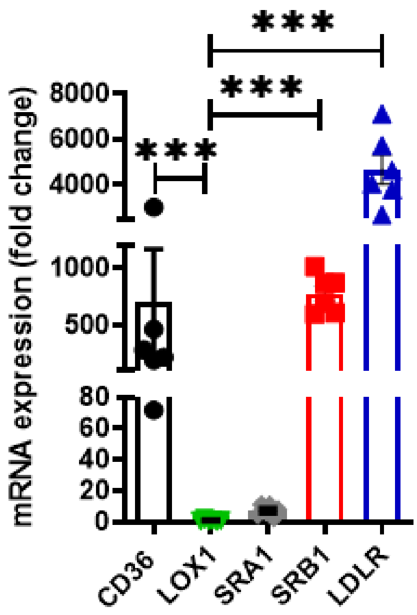

Supplementary Figure 4. Expression of various LDL receptors in LEC.
